# Evolution and recent advances in antibody–drug conjugate therapy for lung cancer: a comprehensive systematic review based on the Trialtrove database (inception to July 1, 2025)

**DOI:** 10.3389/fonc.2026.1793949

**Published:** 2026-05-20

**Authors:** Ye Chen, Yue Zhang, Chunyan Tian, Zhuying Li, Guanghui Guo

**Affiliations:** 1 First Clinical Medical College, Heilongjiang University of Chinese Medicine, Harbin, China; 2 Department of Respiratory Medicine, The First Affiliated Hospital of Heilongjiang University of Chinese Medicine, Harbin, China; 3 Department of Pulmonary Diseases, Hubei Provincial Hospital of Traditional Chinese Medicine, Wuhan, Hubei, China; 4 Hubei Shizhen Laboratory, Wuhan, Hubei, China

**Keywords:** antibody-drug conjugate, biomarkers, clinical trials, linker, lung cancer, payload

## Abstract

**Background:**

Antibody–drug conjugates (ADCs) are emerging targeted therapies in cancer treatment. They selectively deliver cytotoxic payloads to tumor cells, improving efficacy and reducing toxicity. However, the clinical trial landscape in lung cancer remains unclear. This systematic review analyzes registered ADC trials in lung cancer comprehensively.

**Methods:**

Trials were retrieved from the Trialtrove database from inception to July 1, 2025, focusing on ADC interventions in lung cancer. The inclusion criteria required complete ADC data, excluding observational studies or incomplete records. The analyses covered trial numbers, geography, funding, phases, design, targets, linkers, payloads, and biomarkers. This review follows Preferred Reporting Items for Systematic Reviews and Meta-Analyses guidelines.

**Results:**

A total of 546 global trials were identified, of which 466 were included. The trial registrations grew exponentially from one in 2001 to 106 in 2024. Most trials were single-continent and industry-funded. Early phases predominated. A total of 76 targets were involved, with the top 15 (e.g., trophoblast cell surface antigen 2 (TROP2), 18.07%; human epidermal growth factor receptor 2 (HER2), 17.65%) accounting for 78.15%. The linkers were mainly cleavable protease-dependent. Payloads were dominated by DNA topoisomerase I inhibitors. Biomarkers were featured in 85.84% of trials.

**Conclusion:**

This review highlights the rapid expansion and regional focus of ADC trials in lung cancer, driven by early phases and industry support. Diverse targets emphasize TROP2 and HER2, while biomarkers advance precision medicine from standard testing to personalized regimens. Novel targets and combinations enhance selectivity. Optimized linkers mitigate off-target risks, such as the risk of interstitial lung disease (ILD). This analysis offers researchers comprehensive insights and advocates for strengthened intercontinental collaboration and late-phase trials to improve patient outcomes.

## 1 Introduction

Lung cancer ranks among the leading causes of cancer-related deaths worldwide. Despite ongoing advances in diagnosis and treatment, the survival rates for lung cancer patients remain unsatisfactory (1). A 2025 study reported a global premature cancer death probability of 6.49% (95% uncertainty interval 6.49–6.50, with lung cancer as the primary cause in men and the third in women), but timely interventions can extend the life expectancy (2). Lung cancer is classified into non-small cell lung cancer (NSCLC, ~85%) and small cell lung cancer (SCLC, ~15%). NSCLC subtypes include adenocarcinoma (45%), squamous cell carcinoma (21%), and unclassified forms. NSCLC, which accounts for over 80% of diagnosed lung cancer cases, has a varying prognosis depending on stage: early-stage (I–II) patients typically achieve 5-year survival rates of 60%–90%, while more than half of the cases are diagnosed at advanced stages (III–IV), with 5-year survival rates of only 2%–20% (3). SCLC features poor prognosis, high initial distant metastasis risk, and a 5-year survival rate <7% (4).

Primary treatments for lung cancer include surgery, radiotherapy, chemotherapy, targeted therapy, and immunotherapy. For NSCLC, surgery is the preferred choice for early-stage disease, encompassing wedge resection, lobectomy, pneumonectomy, and sleeve resection, often combined with adjuvant radiotherapy or chemotherapy (5). Radiotherapy serves as adjuvant treatment after surgery or as an alternative for inoperable cases, providing local control in stages I–III (6). Chemotherapy is commonly used in advanced or adjuvant settings, with nanoparticle delivery emerging as a means to reduce toxicity. Targeted therapy exploits specific genetic mutations, including monoclonal antibodies, tyrosine kinase inhibitors (TKIs), mammalian target of rapamycin inhibitors, and Kirsten rat sarcoma viral oncogene homolog (KRAS) G12C inhibitors and is frequently applied in driver-mutated NSCLC. Immunotherapy, such as programmed death-1 (PD-1)/programmed death-ligand 1 (PD-L1) inhibitors and cytotoxic T-lymphocyte-associated protein 4 inhibitors, is often used as first-line treatment. Emerging personalized treatments, including laser therapy, photodynamic therapy, cryosurgery, and electrocautery, are also employed (7).

In contrast, the treatment options for small-cell lung cancer (SCLC) are more limited due to its aggressive nature and tendency for early dissemination. Surgery is rarely performed because of bilateral spread (8), although robotic minimally invasive techniques can reduce surgical risks in selected cases (9). Radiotherapy is typically combined with chemotherapy for limited-stage disease (6). Chemotherapy remains the cornerstone of treatment across all stages. Targeted therapies are rarely effective, while immunotherapy is usually reserved for the post-chemotherapy setting. Emerging personalized treatments such as laser therapy, photodynamic therapy, cryosurgery, and electrocautery may also be considered in selected patients (7).

ADC therapy is an emerging targeted anticancer strategy that links monoclonal antibodies (mAbs) to cytotoxic payloads via linkers. It delivers antitumor drugs precisely to tumor cells via selective mAb binding, reducing systemic toxicity and enhancing chemotherapy efficacy—a hallmark of precision oncology breakthroughs (10). ADCs comprise three core components: antibody, linker, and payload (11). Antibodies are typically humanized immunoglobulin G1mAbs with high affinity and long half-life, targeting tumor-associated antigens such as HER2 or TROP2. Optimal targets are overexpressed on tumor cells but are minimally expressed in normal cells, thereby enhancing cytotoxic selectivity and reducing systemic toxicity. Linkers maintain stable conjugation in circulation but allow intracellular cleavage. They are non-cleavable (e.g., thioether, relying on lysosomal degradation in first-generation ADCs) or cleavable (e.g., peptide, disulfide, hydrazone, using tumor microenvironment pH, enzymes, or redox for release). Ideal designs balance stability to avoid off-target toxicity with efficient drug release in cancer cells. Payloads are potent cytotoxic drugs, such as topoisomerase I inhibitors (e.g., duextecan (DXd) and SN-38, which cause DNA damage) or microtubule inhibitors (e.g., mertansine (DM1) and monomethyl auristatin E (MMAE), which disrupt mitosis). They require stability, membrane permeability, and bystander effects (diffusing to adjacent cells). Ideally, they are non-immunogenic and non-toxic in circulation and effective at sub-nanomolar levels to broaden the therapeutic window via targeted delivery (12). ADCs’ mechanism involves antibody binding to cancer cell surface antigens, inducing receptor-mediated endocytosis, lysosomal linker cleavage, payload release, and apoptosis. Concurrently, they activate immune responses by recruiting natural killer cells and disrupting receptor dimerization, yielding direct killing, bystander effects, and immune activation (13).

ADC technology has developed exponentially over more than a century of research. In the early 1900s, Paul Ehrlich proposed the “magic bullet” concept: agents delivering toxins directly to diseased cells while sparing normal tissue. The first human ADC trial, designed in the 1980s, was withdrawn due to high toxicity and suboptimal efficacy. In 2000, gemtuzumab ozogamicin (anti-cluster of differentiation 33 (CD33)) became the first Food and Drug Administration (FDA)-approved ADC for relapsed/refractory acute myeloid leukemia but was withdrawn in 2010 due to adverse events outweighing benefits. In 2011, brentuximab vedotin (anti-CD30) was approved for relapsed/refractory Hodgkin lymphoma and anaplastic large cell lymphoma. In 2013, ado-trastuzumab emtansine (T-DM1, anti-HER2) was approved for metastatic breast cancer, the first for solid tumors (14). Thereafter (15), ADC development accelerated (16), with multiple approvals in recent years (17).

Currently, five ADCs are approved globally for lung cancer, mainly NSCLC (18) (see **Table 1**): three received FDA approval, and two were from China’s National Medical Products Administration (NMPA). Approvals target antigens such as HER2, TROP2, and the mesenchymal–epithelial transition factor (c-Met) based on objective response rates (ORR). Enhertu was FDA-approved in August 2022 for HER2-mutated metastatic NSCLC post-prior therapy, making it the first ADC for lung cancer and targeting 2%–4% of NSCLC patients. It features a drug-to-antibody ratio of 8 and bystander effects, with an ORR of ~58% and a median progression-free survival (PFS) of 12.4 months; however, monitoring for ILD is essential (19). Telisotuzumab vedotin was FDA-approved in May 2025 for previously treated advanced/metastatic non-squamous NSCLC with high c-Met expression (≥50% 3+ staining tumor cells), the first for wild-type c-Met overexpression in 25%–30% of NSCLC patients. Based on accelerated approval from the LUMINOSITY trial, it achieved an ORR of 35% in patients with platinum-resistant disease; peripheral neuropathy monitoring is required (20). Datopotamab deruxtecan was FDA-approved in June 2025 for epidermal growth factor receptor (EGFR)-mutated advanced/metastatic NSCLC post-EGFR TKI and platinum chemotherapy, the first TROP2-directed ADC for this indication and second-line for ~40% of Asian NSCLC patients. Being biomarker-independent, it showed an ORR of 42.5% in TROPION-Lung05/01 trials, with DXd payload outperforming traditional chemotherapy; however, higher ILD rates necessitate monitoring (21). Sacituzumab govitecan received NMPA approval in March 2025 for EGFR-mutated advanced/metastatic non-squamous NSCLC after EGFR inhibitor therapy, making it China’s first TROP2-targeted ADC. Its belotecan-derived payload improves stability, yielding an ORR of 44% in the OptiTROP-Lung03 trial, making it ideal for EGFR-mutated Asian populations with lower toxicity and greater combination potential (22). Trastuzumab rezetecan received NMPA approval in May 2025 for advanced HER2-mutated NSCLC, making it China’s first domestically developed HER2-directed ADC. Similar to Enhertu with an optimized payload, it achieved an ORR of ~50%, offering improved accessibility and tolerability (23). Overall, these ADCs demonstrate the potential of biomarker-guided targeting to overcome resistance, setting a paradigm for advanced lung cancer management (12).

**Table 1:** Currently approved ADCs for the treatment of lung cancer.

Drug	Company	Trade name	Target antigen	Linker	Payload	Approved countries
Trastuzumab deruxtecan	Daiichi Sankyo/AstraZeneca	Enhertu	HER2	Cleavable tetrapeptide-based	DXd (topoisomerase I inhibitor)	FDA, PMDA
Telisotuzumab vedotin	AbbVie	Emrelis	c-Met	Cleavable (valine-citrulline)	MMAE (microtubule inhibitor)	FDA
Datopotamab deruxtecan	Daiichi Sankyo/AstraZeneca	Datroway	TROP2	Cleavable tetrapeptide-based	DXd (topoisomerase I inhibitor)	FDA
Sacituzumab tirumotecan	Sichuan Kelun-Biotech	SKB264	TROP2	CL2A (hydrolyzable)	KL610023 (topoisomerase I inhibitor)	NMPA
Trastuzumab rezetecan	Jiangsu Hengrui Pharma	SHR-A1811	HER2	Cleavable	Camptothecin derivative (topoisomerase I inhibitor)	NMPA

Currently, the majority of ADC trials in lung cancer are conducted in the second-line or later settings following progression on targeted therapy or immunotherapy. This reflects the urgent clinical need for effective salvage options in pretreated patients (24). Compared to other therapies, ADCs offer superior targeting specificity, sustained efficacy, and resistance overcoming, widening therapeutic windows for refractory lung cancer. Despite ADCs’ potential in lung cancer treatment, challenges such as resistance in refractory cases and toxicities (e.g., ILD) limit their clinical use, requiring a systematic analysis of trends and the development of optimization strategies (25). Thus, in-depth analysis of ADC mechanisms, trial outcomes, and limitations in lung cancer is crucial. Ongoing research and trials are expanding the understanding of ADC therapy, addressing challenges to realizing its full potential in lung cancer. The Trialtrove database, which aggregates global trials, provides a robust platform to assess advances in ADCs (26). Accessing recent trial data from Trialtrove enables the evaluation of ADC efficacy, safety, and progression across phases. This analysis provides a thorough understanding of ADC therapeutic potential and evidence-based insights for future research on clinical applications and outcomes. This systematic review addresses the research question using the population, intervention, comparison, outcome, and study design framework: population—lung cancer patients (NSCLC and SCLC); intervention—ADC therapies; comparators—not applicable (review of registered trials); outcomes—trial characteristics, growth trends, geography, funding, phases, designs, indications, mechanisms, targets, linkers, payloads, and biomarkers; study designs—registered clinical trials from Trialtrove.

## 2 Methods

### 2.1 Data sources and selection criteria

Systematic review protocol: This review follows the Preferred Reporting Items for Systematic Reviews and Meta-Analyses (PRISMA) 2020 guidelines for reporting systematic reviews. No formal protocol was pre-registered, but methods were predefined to minimize bias. This study utilized data from the Trialtrove database, which aggregates global clinical trial information. We searched for all trials registered up to July 1, 2025, related to ADC-targeted therapy for lung cancer, using the following search terms: disease tumor: “(lung, non-small cell) or (lung, small cell)” AND drug type: “biological > protein > antibody > antibody-drug conjugate”.

### 2.2 Inclusion and exclusion criteria

Clearly defined inclusion and exclusion criteria guided the selection process in this study. The inclusion criteria encompassed trials explicitly focused on ADC-targeted therapy, with clearly stated therapeutic objectives or mechanisms of action related to lung cancer treatment. The exclusion criteria were more nuanced, targeting trials lacking key information (e.g., undefined drug targets or mechanisms), those without complete datasets, or those involving non-interventional study designs (e.g., observational studies or registries that do not assess treatment outcomes). Trials with missing or unclear drug targets required additional scrutiny. If a trial lacked sufficient details to determine the therapeutic objective, it was excluded from the final analysis.

### 2.3 Handling incomplete data

Incomplete data were handled systematically. To ensure the robustness of our analysis, trials missing substantial information were excluded. If a trial had unknown or unclear drug targets, those targets were excluded unless further cross-referencing with external databases or publicly available open sources clarified the therapeutic approach. This method minimized bias and maintained the integrity of the dataset during analysis.

### 2.4 Data extraction and statistical analysis

Data were extracted independently by two reviewers (YC and YZ) using a standardized form that included the following fields: trial ID, start date, geography, funding, phase, status, design, indications, mechanisms, targets, linkers, payloads, and biomarkers. Disagreements were resolved by consensus with a third reviewer (ZL).

### 2.5 Data analysis

Descriptive statistics, including frequencies and percentages, were used to summarize all trial characteristics. No meta-analysis was performed owing to substantial clinical and methodological heterogeneity among the included trials. As this study is a review of trial registrations rather than published results, a formal risk-of-bias assessment was not conducted; however, potential biases (e.g., reporting bias within the database) are addressed in the limitations section.

## 3 Results

### 3.1 Trial characteristics and funding sources

**Figure 1:**
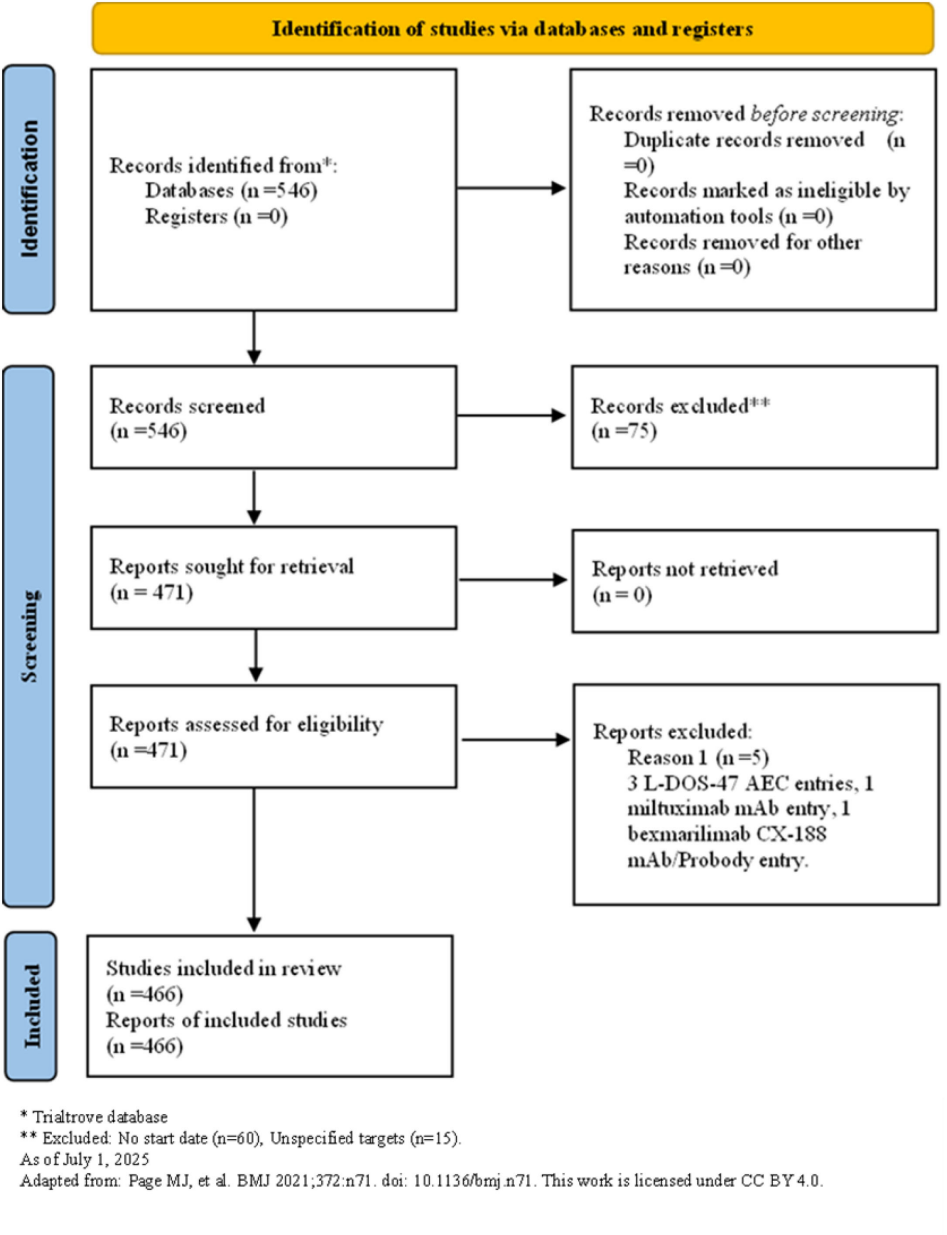
Flowchart of this study.

**Figure 2:**
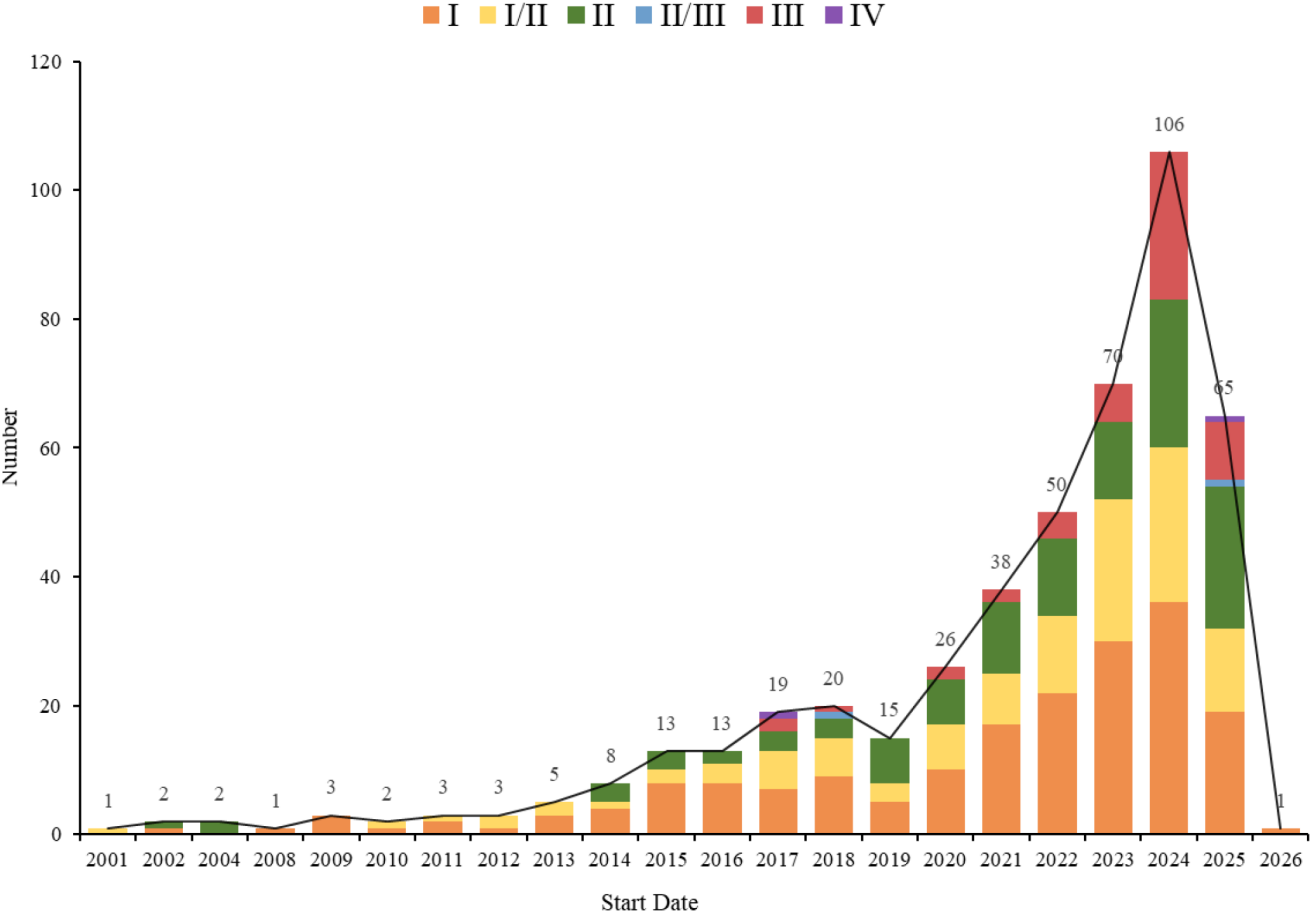
Start date distribution of clinical trials for ADC therapy in lung cancer treatment.

**Figure 3:**
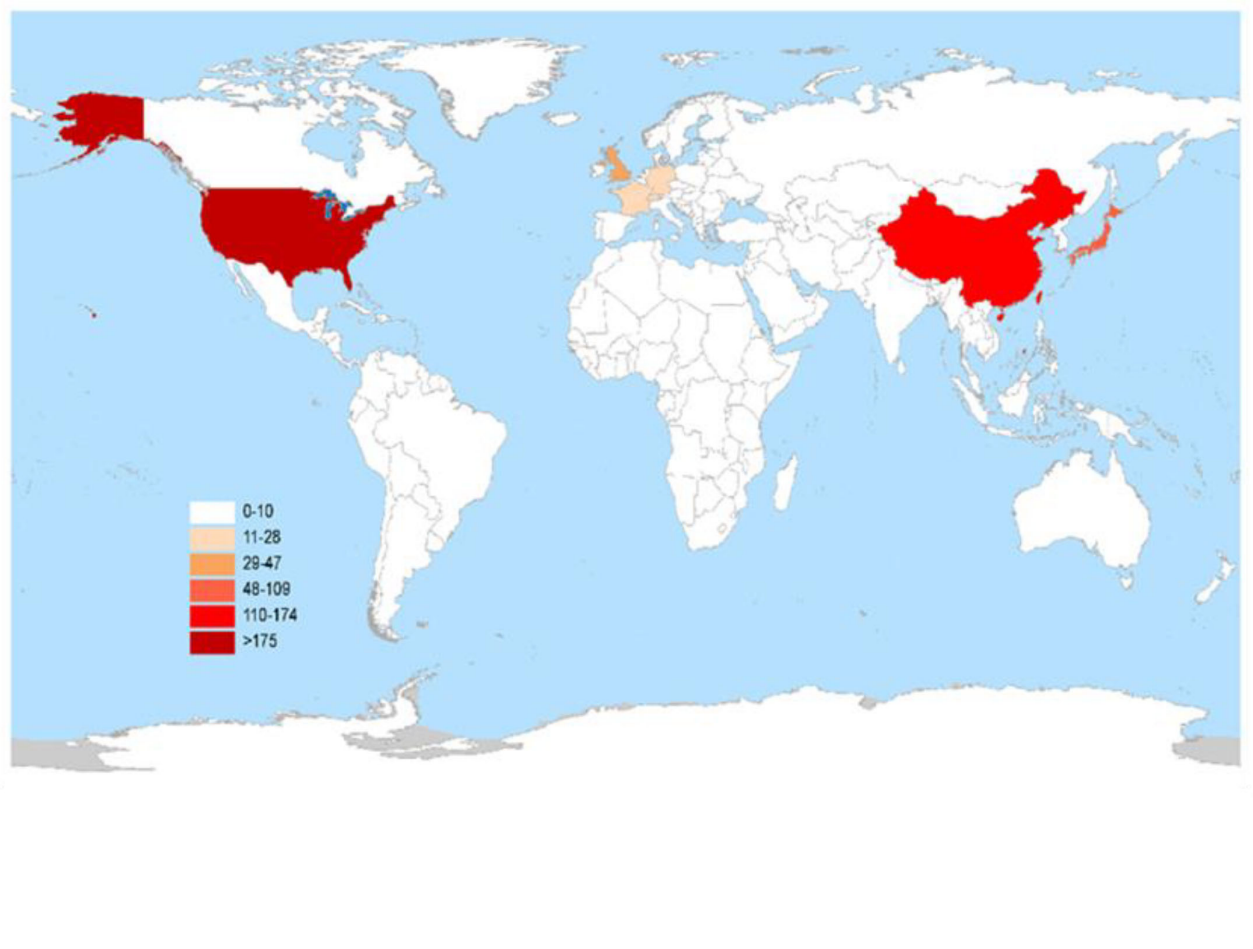
Global distribution of clinical trials for ADC therapy in lung cancer treatment.

**Figure 4:**
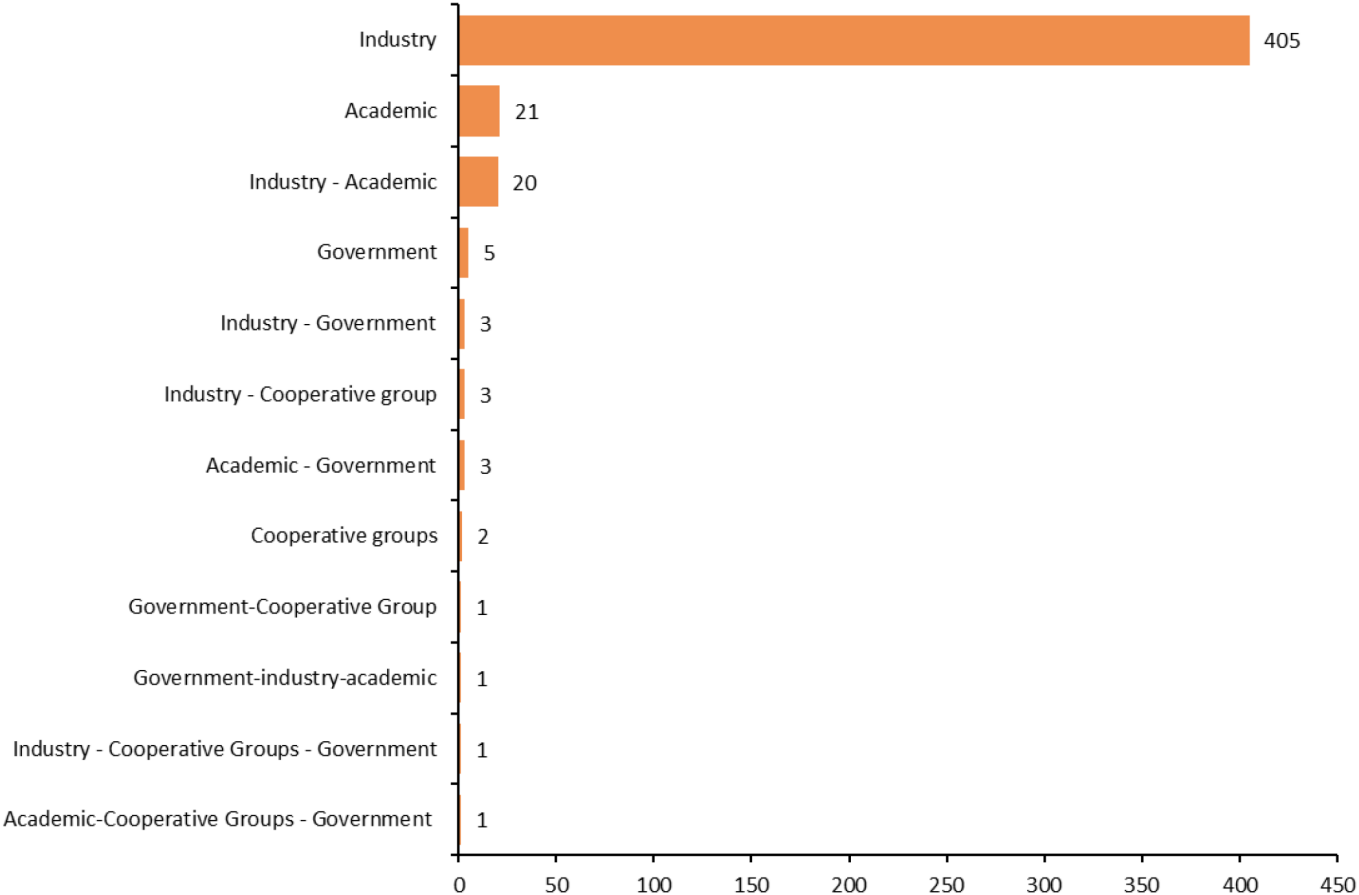
Funding types in research projects.

As of July 1, 2025, a total of 546 clinical trials targeting ADC therapy for lung cancer were registered globally. The excluded trials included 60 lacking specific start dates, 0 in “other” phases, 0 not containing ADC test drugs, and 15 with “unspecified” unknown target drugs, leaving 471 trials for analysis. Subsequently, through a cross-target comparison, five non-ADC data entries were further removed (including three L-DOS-47 AEC entries, one miltuximab mAb entry, and one bexmarilimab CX-188 mAb/Probody entry) to ensure the accuracy and consistency of the analysis, resulting in a total of 466 remaining trials (**Figure 1**). The trial registrations increased gradually from one in 2001 to 106 in 2024, reaching 65 in the first half of 2025, with one planned for 2026 (**Figure 2**). Geographically, most trials were conducted on a single continent, with Asia hosting 166 trials (35.62%), North America 137 trials (29.4%), and Europe 50 trials (10.73%), with a single-continent cumulative proportion at 75.75%; collaborations across two continents included 24 between Europe and Asia (5.15%), 23 between North America and Asia (4.94%), and one between North America and Oceania (0.21%); collaborations across three continents accounted for 11 trials (2.36%), with 33 trials (7.08%) not specifying particular countries (**Figure 3**). The funding sources varied: 405 trials (86.91%) were industry-funded, 21 (4.51%) academically funded, five (1.07%) government-funded, and two (0.43%) funded by collaborative groups; additionally, 20 (4.29%) were jointly funded by industry and academia, three (0.64%) by industry and government, three (0.64%) by industry and collaborative groups, three (0.64%) by government and academia, one (0.21%) by government and collaborative groups, one (0.21%) by government, industry, and academia, one (0.21%) by industry, collaborative groups, and government, and one (0.21%) by academia, collaborative groups, and government (**Figure 4**). Assessment of risk of bias: As this review analyzes trial registrations, risks include incomplete reporting (addressed by exclusion) and industry funding bias (86.91% industry-funded, potentially favoring positive designs).

### 3.2 Clinical trial phases and designs

**Figure 5:**
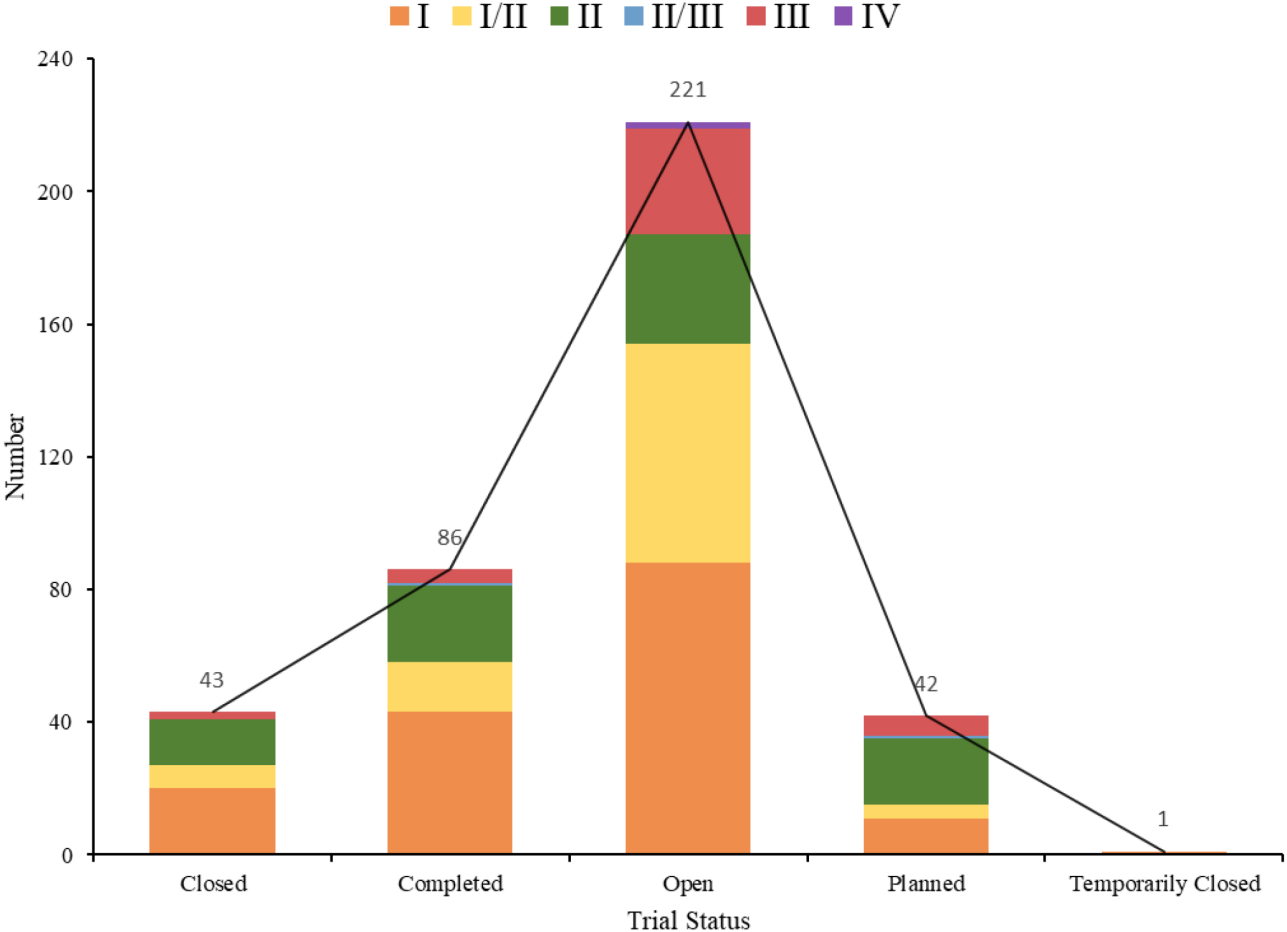
Trial status distribution of clinical trials for ADC therapy in lung cancer treatment.

**Figure 6:**
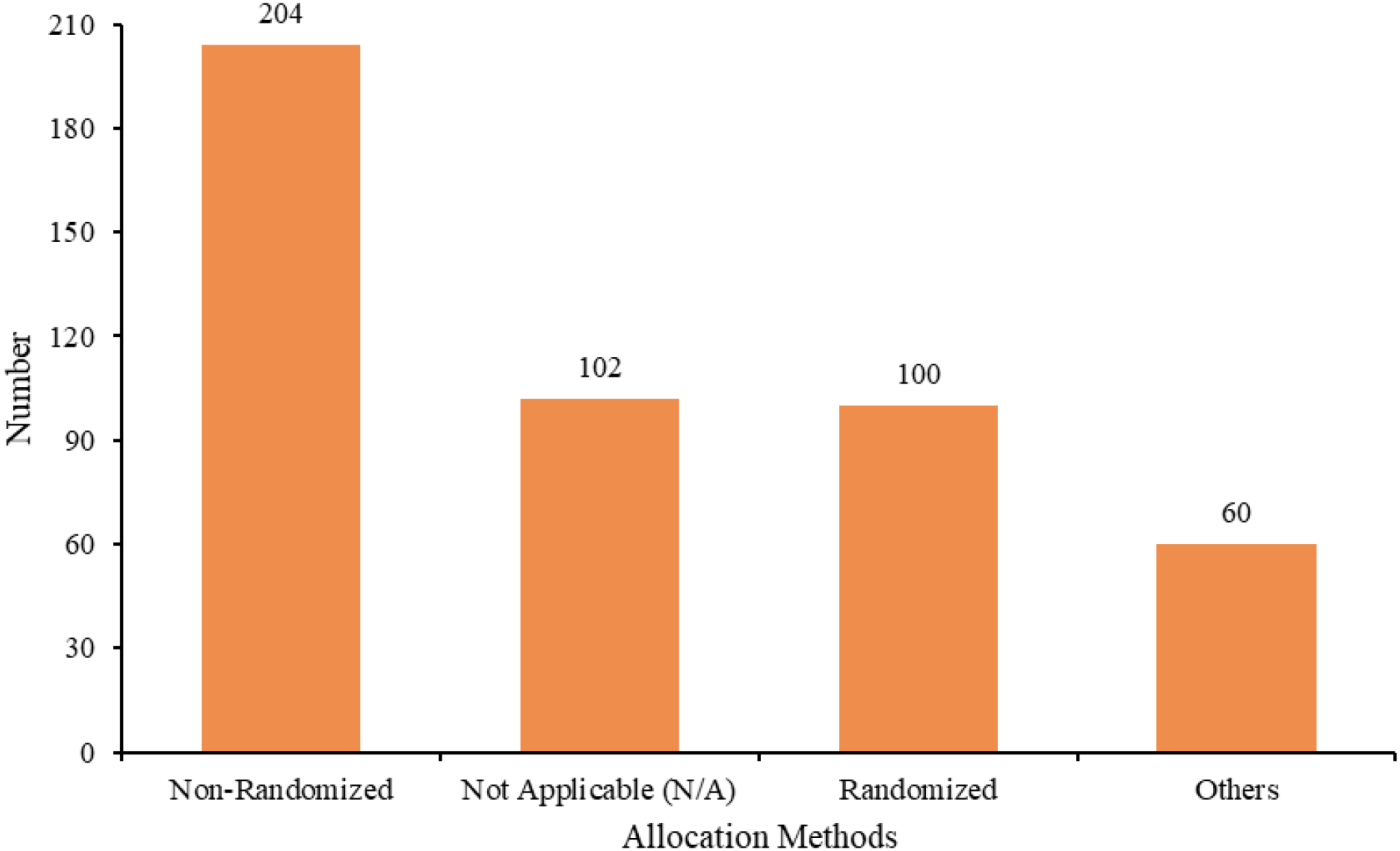
Distribution of allocation methods in clinical trials of ADC for lung cancer treatment.

**Figure 7:**
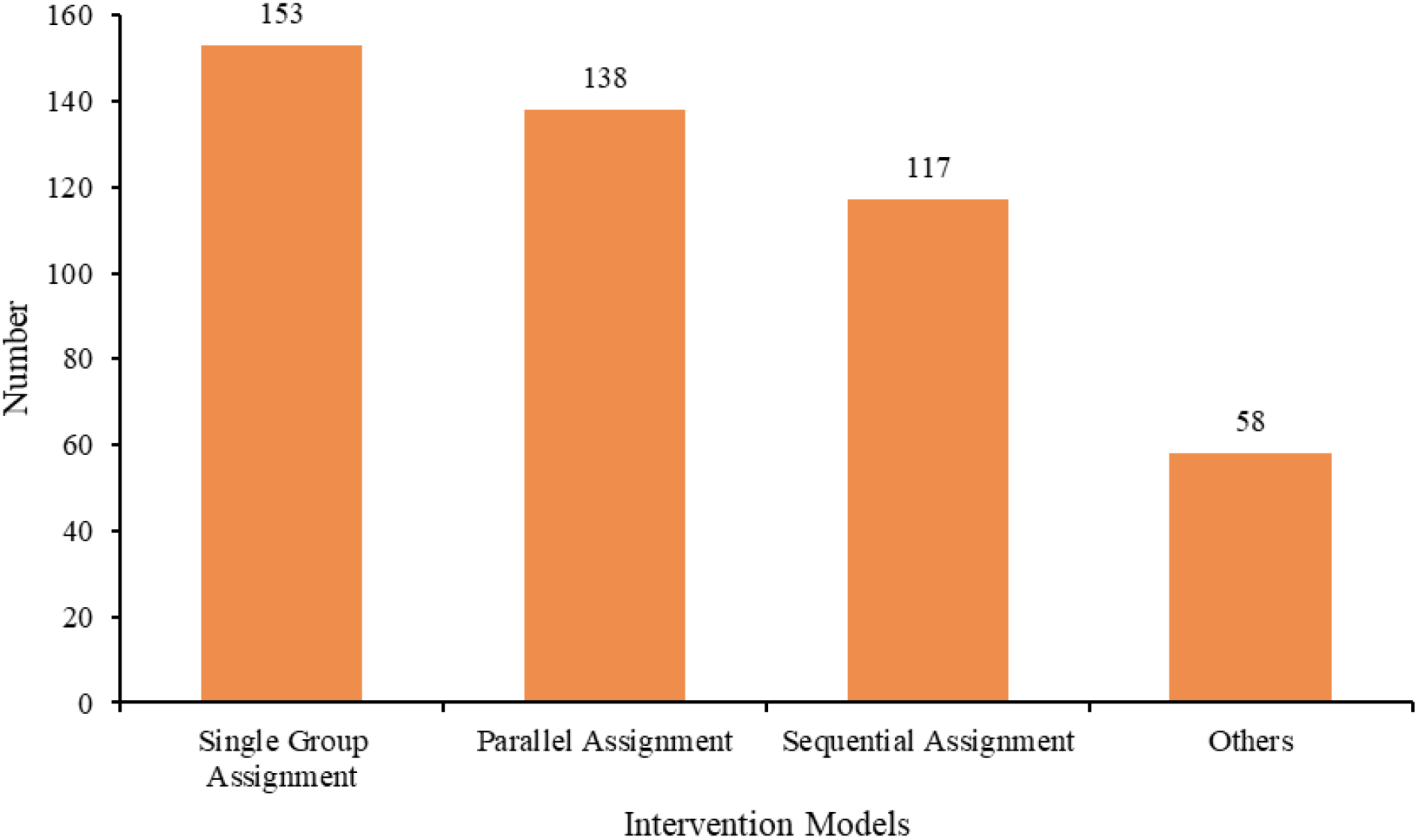
Distribution of intervention methods in clinical trials of ADC for lung cancer treatment.

**Figure 8:**
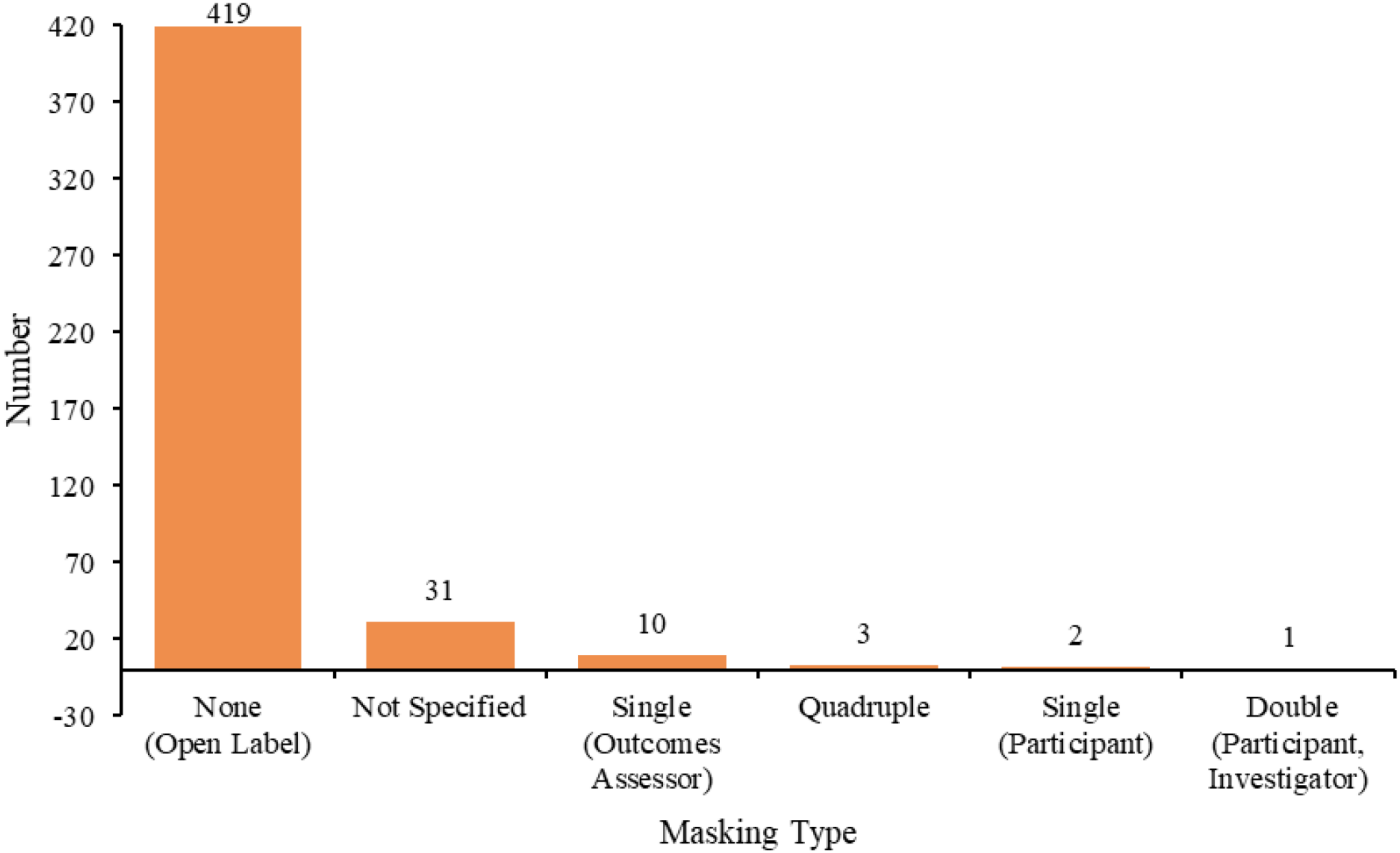
Masking distribution of clinical trials for ADC therapy in lung cancer treatment.

Among all study trial phases, 188 were in phase I, 114 in phase I/II, 111 in phase II, two in phase II/III, 49 in phase III, and 2 in phase IV. The respective trial status varied: 221 trials (47.42%) were in open status, 86 (18.45%) in completed phase, 73 (15.67%) in terminated phase, 43 (9.23%) in planned phase, 42 (9.01%) in closed phase, and one (0.21%) trial in temporarily closed phase (**Figure 5**). The allocation methods were as follows: 204 (43.78%) were non-randomized trials, 102 (21.89%) were not applicable (not applicable), 100 (21.46%) were randomized trials, and 60 (12.88%) were not specified (**Figure 6**). In intervention models, 153 (32.83%) were single-group assignments, 138 (29.61%) were parallel-group assignments, 117 (25.11%) were sequential-group assignments, and 58 (12.45%) were not specified (**Figure 7**). The blinding distribution was as follows: masking: none (open label) had the highest proportion, with 419 (89.91%); not specified were 31 (6.65%); masking: single-blind (outcome assessor) were 10 (2.15%); quadruple-blind (participant, care provider, investigator, outcome assessor) were three (0.64%); masking: single-blind (participant) were two (0.43%); and masking: double-masked (participant, investigator) was one (0.21%) (**Figure 8**).

### 3.3 Clinical indications and mechanism of action analysis

**Figure 9:**
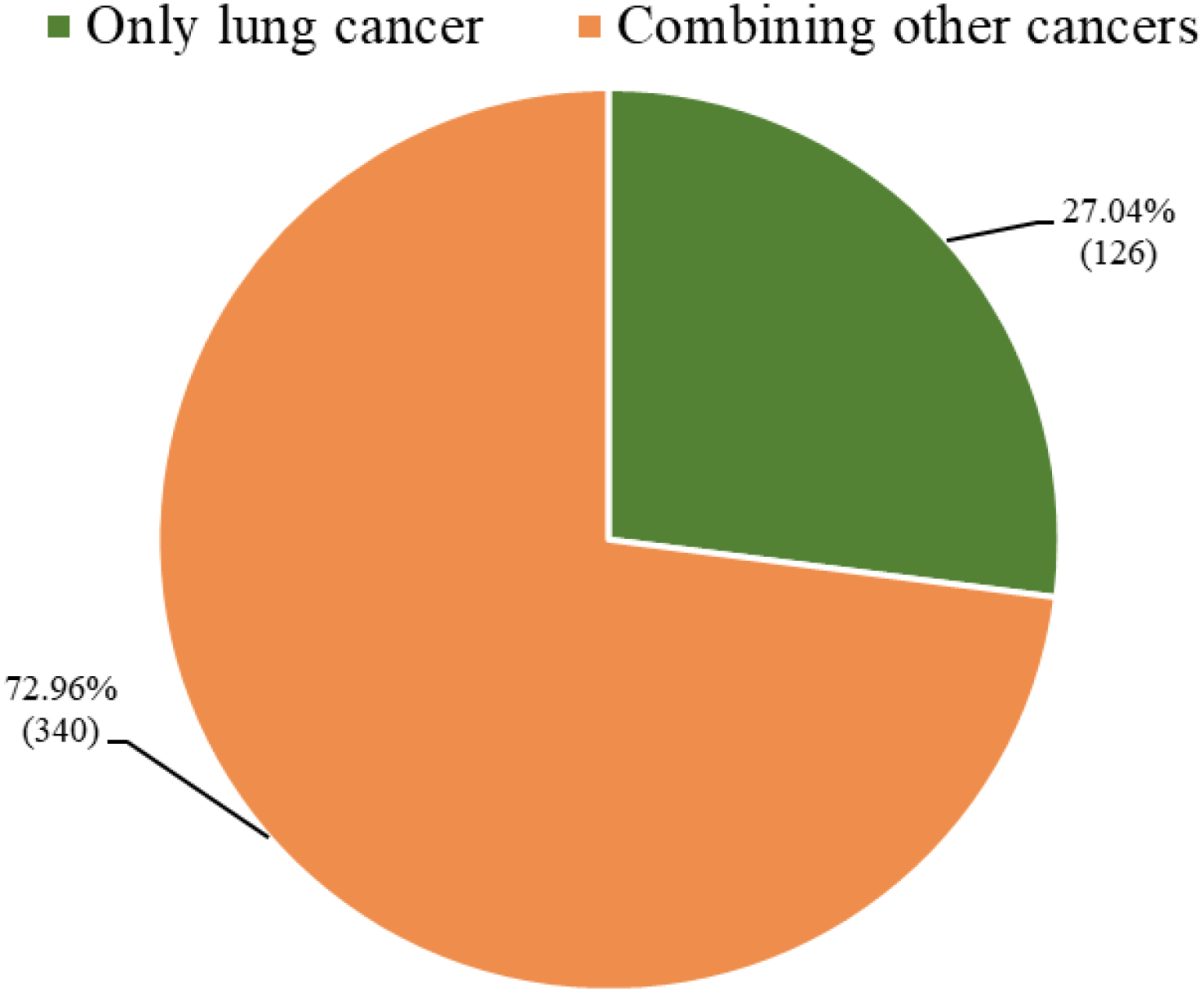
Distribution of indications in clinical trials of ADC for lung cancer.

**Figure 10:**
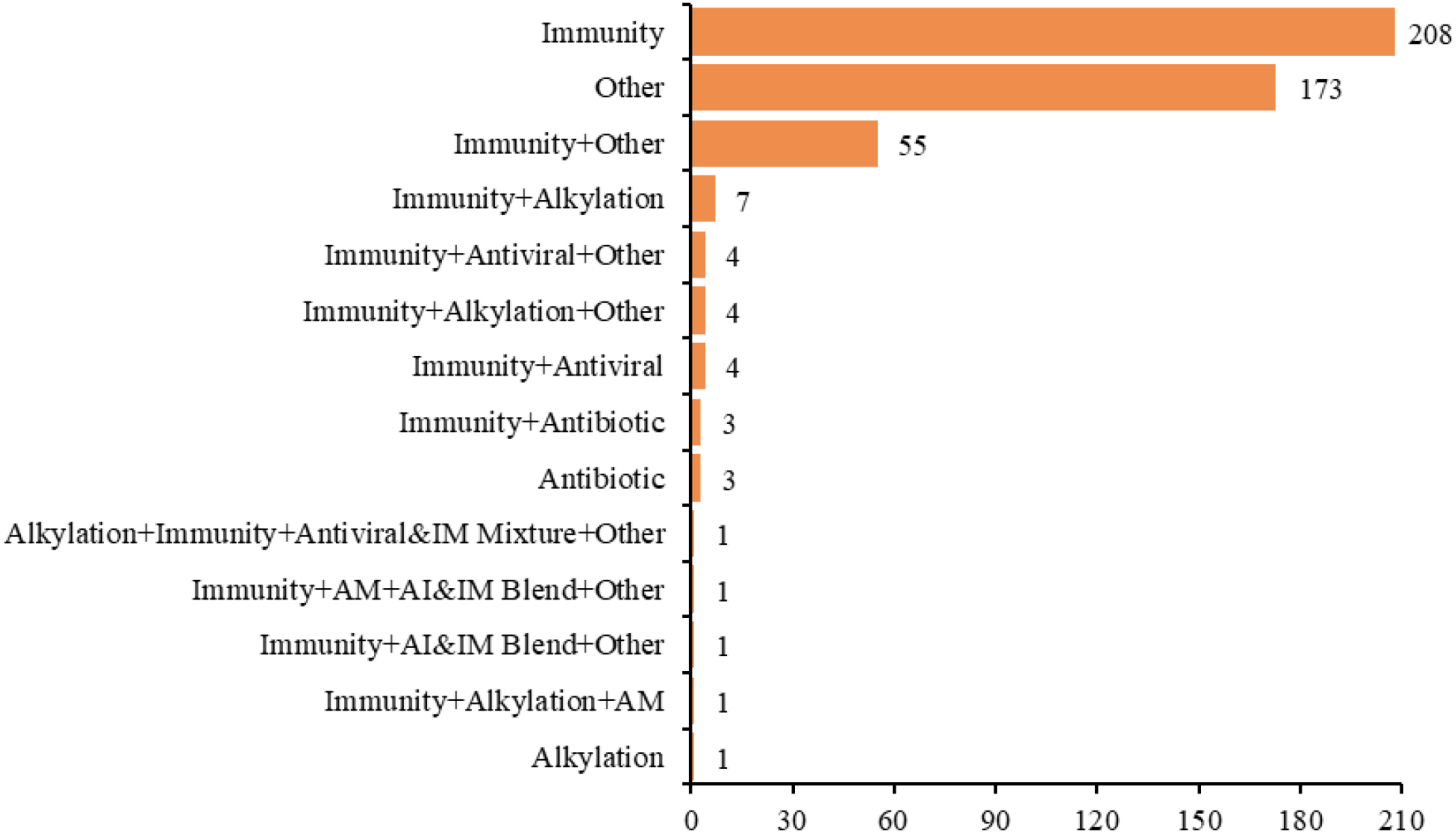
Distribution of drug treatment mechanisms in clinical trials of ADC for lung cancer.

In the ongoing development of research on ADC therapy for lung cancer, the analysis of the 466 registered trials determined that 126 trials (27.04%) were exclusive to lung cancer, with all analyzed projects being NSCLC; the other 340 trials (72.96%) targeted indications combining lung cancer with other malignant tumors (**Figure 9**). The mechanisms of action for ADC drugs tested in clinical settings varied: the immune category had 208 trials (44.64%), the other categories had 173 (37.12%), the antibiotic category had three (0.64%), the alkylating category had one (0.21%), immune combined with other categories had 55 (11.80%), immune combined with alkylating had seven (1.50%), immune combined with antiviral had four (0.86%), immune combined with antibiotic had three (0.64%), immune combined with alkylating and other had four (0.86%), immune combined with antiviral and other had four (0.86%), immune combined with alkylating and antimetabolite had one (0.21%), immune combined with anti-inflammatory and immunomodulatory mixture and other had one (0.21%), immune combined with antimetabolite and anti-inflammatory and immunomodulatory mixture and other had one (0.21%), and alkylating combined with immune and antiviral and immunomodulatory mixture and other had one (0.21%) (**Figure 10**).

### 3.4 Distribution and analysis of targets, linkers, and payloads

In this study, 466 clinical trials involved a total of 476 targets (one trial evaluating three ADCs, eight evaluating two ADCs, and the rest evaluating one ADC each), ultimately identifying 76 unique targets. The distribution of these targets was highly concentrated, with the top 15 targets covering 372 trials (78.15%). Among them, TROP2 was the most common target (86 trials, 18.07%), followed by HER2 (84, 17.65%), B7 homolog 3 (B7-H3): (36, 7.56%), c-Met (23, 4.83%), human epidermal growth factor receptor 3 (HER3) (18, 3.78%), Delta-Like Ligand 3 (DLL3) (17, 3.57%), Nectin-4 (17, 3.57%), EGFR (16, 3.36%), EGFR/HER3 (16, 3.36%), FRα (15, 3.15%), carcinoembryonic antigen-related cell adhesion molecule 5 (CEACAM5) (12, 2.52%), Mesothelin (12, 2.52%), trophoblast glycoprotein (5T4) (7, 1.47%), sodium-dependent phosphate transporter 2B (NaPi2b), and PD-L1 (6, 1.26%). The remaining 61 targets were classified as “other” (104 trials, 21.85%).

Similarly, the study analyzed 476 linkers (with the same distribution as above), classifying them to show the following: cleavable protease-dependent linkers as the dominant type (360, 75.63%), followed by cleavable pH-dependent linkers (45, 9.45%), non-cleavable lysosome degradation-dependent linkers (35, 7.35%), cleavable reduction-dependent linkers (28, 5.88%), cleavable β-glucuronidase-dependent linkers (4, 0.84%), non-cleavable chelation-dependent linkers (2, 0.42%), non-cleavable stable coupling-dependent linkers (1, 0.21%), and cleavable esterase-dependent linkers (1, 0.21%).

**Figure 11:**
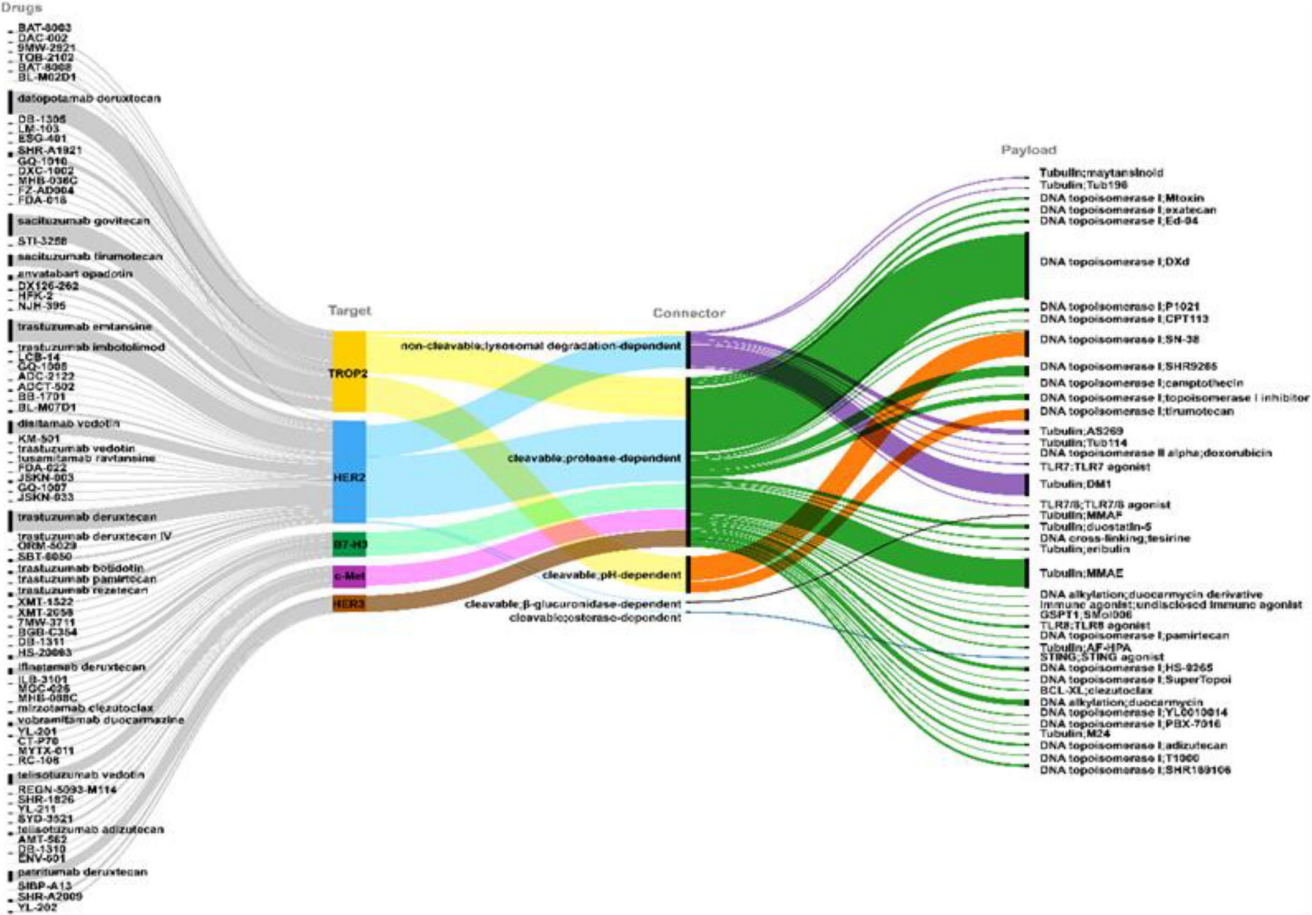
Target-linker-payload architecture of ADC in lung cancer.

Furthermore, the classification analysis of these payloads indicated the following: DNA topoisomerase I inhibitors dominated (261, 54.83%), comprising 30 subclasses, with DXd (68) and SN-38 (24) as the main representatives; followed by tubulin inhibitors (168, 35.29%), comprising 19 subclasses, with MMAE (82) and DM4 (24) as dominant; DNA cross-linking agents were 15 (3.15%), comprising four subclasses, with tesirine (11) as dominant; and DNA alkylating agents were 9 (1.89%), comprising three subclasses, with duocarmycin (7) as dominant. Other categories included DNA topoisomerase II alpha inhibitors (4, 0.84%) as well as emerging mechanisms such as Toll-like receptor agonists and STING agonists (each accounting for 0.42%–0.63%) (**Figure 11**).

### 3.5 Clinical stage, treatment setting, response rates, and survival outcomes

The majority of the 466 included trials focused on advanced or metastatic stages (stage III/IV, advanced, metastatic, extensive, locally advanced), accounting for 95.06% (443/466). At the same time, a substantial proportion also targeted limited-stage or early-stage disease (stage I/II, early-stage, limited-stage, resectable, neoadjuvant), accounting for 90.13% (420/466), with some overlap in stage descriptions. Treatment settings were predominantly second-line (68.67%, following prior immunotherapy or platinum-based chemotherapy), followed by first-line (21.46%) and adjuvant/neoadjuvant/maintenance settings (9.87%).

ORR was listed as a primary or secondary endpoint in 88.41% (412/466) of all trials. When stratified by the top 15 targets (in descending order of frequency), the proportion of trials that included ORR as an endpoint was as follows: TROP2—90.70%, HER2—90.48%, B7-H3—88.89%, c-Met—91.30%, HER3—88.89%, DLL3—88.24%, Nectin-4—88.24%, EGFR—87.50%, EGFR/HER3—87.50%, FRα—86.67%, CEACAM5—83.33%,—Mesothelin 83.33%, 5T4—85.71%, NaPi2b—85.71%, and PD-L1—83.33%. PFS was listed as a key efficacy endpoint in 98.28% (458/466) of trials, with overall survival (OS) in 96.99% (452/466) of trials. Data were compiled from the Trialtrove database.

### 3.6 Clinical biomarker prediction

In the global-industry-initiated clinical trials for ADC therapy in lung cancer, 85.84% (i.e., 399 trials) of the conducted studies were on tumor biomarkers. The common uses of these biomarkers, classified by combinations, are as follows: the combination of diagnosis, prediction, induction, and prognosis involved 233 trials; companion diagnosis, diagnosis, prediction, induction, and prognosis combination involved 114 trials; diagnosis, prediction, and prognosis combination involved nine trials; diagnosis, early detection, prediction, induction, prognosis, and risk assessment combination involved eight trials; companion diagnosis, diagnosis, early detection, prediction, induction, prognosis, and risk assessment combination involved eight trials; diagnosis and prognosis combination involved seven trials; induction and prognosis combination involved six trials; diagnosis, induction, and prognosis combination involved six trials; prediction, induction, and prognosis combination involved five trials; and sole prognosis use involved three trials. Additionally, by individual uses, prognosis use involved 399 trials, induction use involved 380 trials, diagnosis use involved 377 trials, prediction use involved 377 trials, companion diagnosis use involved 122 trials, early detection use involved 16 trials, and risk assessment use involved 16 trials.

## 4 Discussion

In recent years, ADC therapy for lung cancer has gained considerable attention and made notable progress. Trial registrations started low in 2001, began to increase after 2013, accelerated markedly from 2015 onward, and peaked at 106 trials in 2024. This surge has been accompanied by a substantial increase in research funding and investment over the past decade. This upward trend not only reflects growing scientific interest but also demonstrates increasing confidence in the therapeutic potential of ADCs for lung cancer. As the field continues to evolve, a deeper understanding of key efficacy determinants—such as antigen expression, heterogeneity, trial design, tumor microenvironment, resistance mechanisms, patient-specific factors, and pharmacokinetics profiles—has become essential for optimizing the future (27). Building upon these global trends, the regional distribution of trials further reveals significant imbalances and disparities (28).

The majority of trials are concentrated in North America and Asia, with substantially fewer conducted in Europe. This geographic pattern reflects differences in regional epidemiology, mutation prevalence, and resource allocation (29)—for example, the high prevalence of EGFR mutations in Asian patients has driven the rapid development of HER2- or TROP2-targeted ADC trials to optimize outcomes in NSCLC. However, prior treatment with EGFR TKIs frequently leads to resistance mutations (e.g., L858R, T790M), thereby increasing the risk of secondary resistance (30). In contrast, North American patients with NSCLC exhibit higher KRAS mutation rates and lower EGFR mutation rates, which significantly influence target selection and therapeutic strategies. These genetic heterogeneities underscore the need to account for population-specific variations to ensure consistent efficacy across diverse patient populations (31). Racial and ethnic differences in mutation profiles and treatment responses further underscore the need for diverse patient recruitment in clinical trials to reduce health disparities and improve the generalizability of findings. Single-continent trials accounted for 75.75% of all studies. In contrast, multicontinent collaborations enable broader ethnic integration, reduced selection bias, cross-validation of results, and smoother regulatory approvals (31). Ultimately, enrollment of diverse populations provides critical insights into genetic influences on treatment responses, which is essential for developing truly personalized ADC therapies tailored to global patient needs.

Funding sources exhibit clear diversity, supporting balanced regional development and reflecting extensive international collaboration. Industry sponsors primarily focus on commercialization, whereas government and academic sponsors emphasize innovation despite resource limitations. This complementary synergy effectively balances practical development with exploratory research. Consequently, industry-sponsored trials often report more favorable results. In contrast, academic and government-funded trials tend to present more neutral outcomes, potentially attributable to differences in reporting practices or conflicts of interest (32). From a global perspective, core funders such as government agencies have increasingly committed to strengthening clinical trial standards, including rigorous data management and greater patient involvement, reflecting a broader emphasis on quality improvement. Furthermore, the distribution of clinical design phases clearly illustrates the pivotal role of funding sources in advancing ADC trials to later stages. The current status of ongoing trials highlights the highly dynamic nature of ADC research in lung cancer. Completed trials provide valuable insights into efficacy and safety. At the same time, the large number of ongoing and planned studies demonstrates strong momentum and the field’s ability to overcome key barriers, such as patient recruitment and demonstrating efficacy. Early-phase (phase I and I/II) trials predominate, reflecting the critical need for initial safety and dose-finding studies, whereas late-phase (phase III) trials remain limited primarily due to toxicity and efficacy challenges (33). Recent literature attributes the predominance of phase I/II trials to the need for rigorous payload safety assessment, while the scarcity of phase III trials is largely due to terminations caused by serious adverse events, most notably ILD. In addition, intrinsic resistance mechanisms and tumor heterogeneity frequently delay progression from early to late phases, underscoring the urgent need for rational combination strategies to generate robust clinical evidence (34).

The predominance of open status trials is closely linked to the post-2019 acceleration in ADC development, particularly for innovative candidates targeting novel antigens or employing bispecific designs, which are actively driving patient recruitment. Regulatory requirements to maintain active enrollment further contribute to the high proportion of open trials (35). Completed trials are predominantly early-phase (I/II) studies that have already yielded results supporting regulatory approvals or subsequent expansion cohorts. The completion rates reflect meaningful successes in early development, although the lack of standardized biomarkers continues to hinder comprehensive efficacy analysis (26). Termination status clearly reflects the persistent challenges in safety and toxicity management—for example, multiple phase III trials of rovalpituzumab tesirine (targeting DLL3) were terminated due to inferior OS and PFS (median OS 6.3 months vs. 8.6 months) (36), while telisotuzumab vedotin (c-Met) and ABBV-399 programs were halted primarily because of severe adverse reactions, low ORR, and insufficient efficacy. These toxicities largely stem from off-target effects of the cytotoxic payload (e.g., DM1, DXd) and suboptimal dose optimization (37). The literature consistently identifies toxicity and insufficient efficacy as the primary drivers of trial termination, underscoring the critical need for continued ADC structural optimization (38). Nevertheless, sustained investment continues to drive new trial planning, particularly for cutting-edge designs such as novel bispecific ADCs (39). These advances are expected to enhance target specificity and reduce off-target effects, paving the way for greater clinical success in the coming years. Closed status trials typically result from successful cohort expansion following preliminary efficacy and safety reviews.

The distribution of blinding practices reveals notable deficiencies, largely attributable to the dominance of early-phase trials and the inherent toxicity challenges posed by ADC agents. This lack of blinding increases the risk of performance and detection bias, reduces the reliability of efficacy outcomes, and ultimately delays the translation of promising agents into clinical practice. These limitations are closely linked to recruitment challenges arising from patient–investigator mismatches and the practical difficulties of implementing blinding in trials involving highly toxic agents (40). Establishing a robust feedback loop between preclinical research, clinical results, and iterative progress is therefore crucial for the continued advancement of ADC development, ultimately offering new hope to patients with limited treatment options.

In ADC trials, a clear indication bias is evident: only 27.04% are lung-cancer-exclusive, while the majority employ “basket” designs. Although this approach accelerates drug development across tumor types, it fragments disease-specific evidence and hinders the formulation of lung-cancer-specific clinical guidelines. NSCLC overwhelmingly dominates the landscape, with virtually no dedicated trials for SCLC, largely due to differences in target expression, aggressive biology, and limited biomarkers of SCLC, thereby exacerbating existing therapeutic disparities (36). Immune mechanisms predominate among the tested ADCs, with many incorporating PD-1/PD-L1 inhibitors to enhance antitumor responses and overcome resistance via synergistic payload effects. Given the marked molecular heterogeneity of lung cancer, standardization of trial designs, regimens, and biomarker criteria is urgently needed to reduce toxicity, improve outcome consistency, and accelerate successful clinical translation. This need extends to the structural optimization of ADCs, where the choice of target, linker, and payloads ultimately determines therapeutic precision, toxicity risk, and applicability across heterogeneous lung cancer subtypes (41).

In ADC trials, target selection is fundamental: surface antigens enable precise payload delivery, minimize off-target toxicity, and enhance the therapeutic index (42). The present analysis reveals a highly concentrated target distribution. TROP2 is the most common target, with low expression in normal tissues but high overexpression in solid cancers, including epithelial tumors (colon, pancreatic, breast), where it plays a key role in proliferation, migration, invasion, and metastasis (43). It is overexpressed in approximately 60% of squamous cell carcinomas and 42%–64% of adenocarcinoma (44). Two TROP2-targeted ADCs have been approved: datopotamab deruxtecan and sacituzumab tirumotecan. Early trials demonstrated promising activity and manageable safety of datopotamab in pretreated patients (45). In the TROPION-Lung01trial, Dato-DXd significantly improved PFS (4.4 vs. 3.7 months, hazard ratio (HR) = 0.75; *P* = 0.004), ORR (26.4% vs. 12.8%), duration of response DOR (7.1 vs. 5.6), and disease control rate (DCR) (77.3% vs. 64.9%) (46). Recent results highlight sacituzumab govitecan’s potential in patients with resistant NSCLC. In the OptiTROP-Lung04 trial, it improved PFS (8.3 vs. 4.3 months, HR = 0.49; *P* < 0.0001), ORR (60.6% vs. 43.1%), and OS (HR = 0.60; *P* = 0.001) (22). Multiple dual-target ADCs (e.g., HER3/TROP2, EGFR/TROP2) are under investigation to overcome heterogeneity, emphasizing TROP2 as a key biomarker for treatment. These findings inform the integration of TROP2 into ADC clinical guidelines and highlight the need for further research on toxicity management and biomarker-driven patient selection.

Beyond TROP2, other targets such as HER2 also demonstrate considerable potential. HER2 is a transmembrane receptor with intrinsic tyrosine kinase activity, encoded by the erb-b2 receptor tyrosine kinase 2 gene. It consists of three main domains: an extracellular ligand-binding domain, a transmembrane domain, and an intracellular kinase domain. Upon dimerization, HER2 activates the PI3K/AKT and MAPK signaling pathways, thereby regulating cell growth, survival, and proliferation (47). Although lowly expressed in normal tissues, HER2 drives tumorigenesis through overexpression or activating mutations in a wide range of tumors, with the highest frequency in bladder cancer, followed by uterine, cervical, colorectal, lung, and breast cancers. In NSCLC, HER2 overexpression occurs in 2%–35% of cases, gene amplification in 2%–20%, and mutations in 1%–4% (predominantly exon 20 insertions) (48). Two HER2-targeted ADCs have received regulatory approval: trastuzumab deruxtecan and trastuzumab rezetecan. In a phase II study of trastuzumab deruxtecan in HER2-overexpressing NSCLC after prior treatment, cohort 1 achieved an ORR of 26.5% (13/49 partial responses), while cohort 1A achieved 34.1% (14/41, including two complete responses and 12 partial responses). These results fill important unmet needs in HER2-positive NSCLC and strongly support further clinical development (49). Trastuzumab deruxtecan has been approved for HER2-mutated NSCLC and shows particular promise in patients with resistant disease. In the HORIZON-01 phase II trial (94 patients), an ORR was reported in 69 patients with a median follow-up of 8.7 months, demonstrating manageable safety and a low incidence of ILD (50). Novel HER2 bispecific ADCs and ADCs conjugated to MMAE are currently under active development. Overall, HER2 represents a highly promising target, and ongoing research is rapidly expanding the therapeutic options for HER2-positive lung cancer (51).

Beyond HER2, B7-H3 has emerged as another highly promising target for ADC development. B7-H3 is a member of the B7 family of immune checkpoints, characterized by transmembrane and immunoglobulin-like domains. It inhibits T-cell activation and promotes immune escape, thereby facilitating tumor progression (52). Although expressed at low levels in normal tissues, B7-H3 is markedly overexpressed in multiple cancers, particularly SCLC, where it is strongly associated with poor prognosis (53). Its tumor-specific expression makes it particularly promising for ADC development, enabling precise payload delivery while minimizing toxicity to normal tissues. Although no B7-H3-targeted ADC has yet been approved, multiple candidates are currently in active clinical development. A leading candidate is ifinatamab deruxtecan, an anti-B7-H3 ADC with a cleavable linker and deruxtecan payload designed for efficient endocytosis and intracellular release. It has demonstrated promising efficacy and acceptable safety in early clinical studies. In the IDeate-Lung01 Phase II trial in pretreated patients with extensive-stage SCLC (including those with brain metastases), doses of 8 and 12 mg/kg were evaluated. At the 12-mg/kg dose, the ORR reached 48.2%, DCR 87.6%, median DOR 5.3 months, PFS 4.9 months, and OS 10.3 months. The most commonly reported adverse events were gastrointestinal and hematologic toxicities (54). The ongoing IDeate-Lung02 Phase III trial is recruiting 540 patients with relapsed SCLC to validate its clinical value (55) further. These studies aim to validate the superiority of B7-H3-targeted ADCs in relapsed SCLC and open new therapeutic avenues for patients with resistance and brain metastases. Notably, B7-H3 frequently co-expresses with PD-L1, which has spurred the development of dual-targeting strategies (e.g., B7-H3/PD-L1) to enhance immune activation and overcome resistance, ultimately improving the overall therapeutic profile (56). Favorable outcomes from these trials are expected to improve prognosis in SCLC, advance next-generation immunotherapies, and expand the scope of precision medicine in this difficult-to-treat disease.

Beyond B7-H3, c-Met is another key target: a tyrosine kinase receptor encoded by the MET gene that, upon binding hepatocyte growth factor, regulates cell proliferation, survival, migration, and invasion. It is expressed at low levels on normal epithelial and endothelial cells, where it plays a physiological role in tissue repair, migration, and development. In cancer, aberrant activation drives tumor growth, angiogenesis, metastasis, and drug resistance, making c-Met an attractive therapeutic target (57). In NSCLC, c-Met overexpression or MET exon 14 skipping mutations occur in approximately 3–4% of cases. It is also a key driver in liver cancer, triple-negative breast cancer, ovarian cancer, and colorectal cancer (57). In the ADC field, telisotuzumab vedotin (an anti-c-Met antibody conjugated to the microtubule inhibitor vedotin) has advanced to late-stage development. In the LUMINOSITY Phase II trial (177 pretreated patients with c-Met-overexpressing NSCLC), telisotuzumab vedotin achieved an ORR of 28.6% overall (34.6% in high expressors and 22.9% in intermediate expressors). The median DOR was 8.3 months (9.0 months in high expressors and 7.2 months in intermediate expressors). Median OS was 14.5 months (14.6 months in high expressors and 14.2 months in intermediate expressors). Median PFS was 5.7 months (5.5 months in high expressors and 6.0 months in intermediate expressors) (58). In a phase I/Ib study (NCT02099058), combining telisotuzumab vedotin with osimertinib in EGFR-mutated, c-Met-overexpressing NSCLC patients after osimertinib progression showed favorable pharmacokinetics similar to monotherapy (59). These trials demonstrate promising activity for dual EGFR/c-Met targeting, broadening precision medicine through multi-kinase inhibition and combination strategies, ultimately benefiting patients with resistant NSCLC.

Beyond c-Met, HER3 ranks as the fifth most frequent target: it is a member of the HER family of receptor tyrosine kinases, similar to EGFR, HER2, and HER4 (60). It is expressed at low levels in normal epithelial and endothelial cells, where it physiologically aids tissue repair, migration, and development. In tumors, HER3 is frequently overexpressed, mutated, or amplified (61). In lung cancer, particularly NSCLC, HER3 expression reaches 83% and is strongly linked to increased metastasis and reduced survival (62). HER3 is also overexpressed in breast, gastric, rectal, and pancreatic cancers, making it an ideal target for cross-tumor ADC development. Although no HER3-targeted ADC has yet been approved, several candidates show strong potential, particularly for EGFR-mutated resistant NSCLC. A key candidate, patritumab deruxtecan, was evaluated in a 2022 phase I study involving 57 patients with advanced EGFR-mutated NSCLC after TKI failure, administered at 5.6 mg/kg every 3 weeks. It achieved an ORR of 39% (95% confidence interval (CI) 26.0–52.4) and median PFS of 8.2 months (95% CI 4.4–8.3), with activity independent of resistance mechanisms and HER3 expression levels (63). The most common grade ≥3 adverse events were hematologic toxicities. These results highlight the broad, mechanism-agnostic activity of HER3-DXd (63). In the 2023 HERTHENA-Lung01 Phase II trial (225 patients post-TKI/platinum), the ORR was 29.8% (95% CI 23.9–36.2), with a median DOR of 6.4 months, PFS of 5.5 months, and OS of 11.9 months (64).In the 2024 U31402-A-U102 Phase I study (102 patients), the ORR was 41.0% (95% CI 30.0–52.7), with median PFS 6.4 months and OS 16.2 months (95% CI 11.2–21.9), identifying erb-b2 receptor tyrosine kinase 3 and topoisomerase I as key resistance markers (65). In the 2025 TUXEDO-3 Phase II cohort 2 (20 patients with brain metastases), with a median follow-up of 5.3 months, the intracranial ORR was 30% (95% CI 11.9–54.3) in non-squamous NSCLC (66). These trials collectively demonstrate that dual-target strategies (e.g., EGFR/HER3, HER3/TROP2, HER3/MUC1) can optimize treatment regimens, and combining HER3-targeted ADCs with checkpoint inhibitors further broadens precision medicine approaches in lung cancer, ultimately benefiting a wider patient population.

Emerging and novel targets are rapidly expanding the therapeutic landscape of ADCs in lung cancer. Beyond the dominant TROP2, HER2, and B7-H3, promising candidates include DLL3 (particularly in relapsed SCLC, with ZL-1310 showing encouraging activity and FDA Fast Track designation), FRα, CEACAM5, and 5T4 (24). These novel targets address unmet needs in both NSCLC and SCLC, especially in patients with resistance or brain metastases. Dual-targeting strategies (e.g., EGFR/HER3, HER3/TROP2) are also gaining momentum to overcome tumor heterogeneity (16). Continued exploration of these emerging targets, combined with optimized linkers and payloads, will be essential to broaden the therapeutic window and improve outcomes across molecular subtypes.

Other important targets include DLL3, which is particularly relevant for relapsed extensive-stage SCLC. ZL-1310, a DLL3-targeted ADC, achieved an ORR of 40% in a phase I study of relapsed ES-SCLC (including patients with brain metastases), with manageable safety and no severe ILD, leading to FDA fast track designation and the initiation of a phase III trial, positioning DLL3 as a validated biomarker (67). Nectin-4 is overexpressed in both NSCLC and SCLC, where it promotes metastasis. ADCs targeting Nectin-4 utilize internalization for efficient payload delivery, with several candidates currently in clinical trials for advanced solid tumors, including lung cancer (68). EGFR-targeted ADCs primarily rely on endocytosis and intracellular payload release in NSCLC. Combining them with immunotherapy has shown potential to overcome resistance, although close monitoring of adverse events remains essential (69). Bispecific ADCs targeting EGFR/HER3, such as BL-B01D1, have shown encouraging activity and safety in advanced solid tumors. In a study of 174 patients, 13 with SCLC demonstrated meaningful responses in patients with heavily pretreated disease (70). Izalontamab, a bispecific ADC targeting EGFR and HER3, demonstrated good tolerability and meaningful activity in a phase I/Ib study involving 60 patients, including 49 with advanced NSCLC (71). ZW191, an FRα-targeted ADC (NCT06555744), is currently under investigation in advanced solid tumors and holds potential for NSCLC (72). Tusamitamab ravtansine, a CEACAM5-targeted ADC, achieved an ORR of 20.3% and a median DOR of 6.7 months in patients with high-CEACAM5-expressing NSCLC (NCT02187848), with a manageable safety profile, indicating potential for further development in lung cancer (73). IXB010, a 5T4-targeted ADC, demonstrated 80%–99% tumor inhibition and good cytotoxicity in 5T4-expressing NSCLC patient-derived xenograft models, with favorable tolerability in a phase I trial (NCT06545331) (74). In gefitinib-resistant NSCLC, the NaPi2b pathway is regulated by miR-124-3p/SLC34A2, which controls cell viability, apoptosis, and metastasis. SLC34A2 promotes tumor growth and metastasis, and the NaPi2b-targeted ADC, lifastuzumab vedotin, has shown promising activity and safety in NSCLC (NCT02124083), suggesting potential to overcome TKI resistance (75). Finally, durvalumab-MMAE, a PD-L1-targeted ADC, achieved >60% tumor inhibition with low toxicity in PD-L1-expressing NSCLC preclinical models, supporting further pharmacokinetic and clinical evaluation (76).

In lung cancer ADCs, linkers play a critical role in regulating stability, pharmacokinetics, and payload release to optimize the therapeutic index within the tumor microenvironment (77). The present analysis reveals a preference for cleavable linkers, predominantly protease-dependent, followed by pH- and reduction-dependent types (78). Protease-dependent linkers enable lysosomal release (e.g., Cathepsin B) (77) with good specificity for the lung microenvironment (79)—for example, valine–citrulline linkers in TROP2- and HER2-targeted ADCs promote internalization and bystander killing (80). However, premature cleavage remains a major drawback, causing ILD at high doses (81). Recent enzyme optimization of datopotamab improved penetration and tolerability, resulting in PFS superior to that with chemotherapy (82). pH-dependent linkers exploit the acidic tumor pH (5–6) for cleavage (e.g., hydrazone or ester) and constitute a notable proportion of lung cancer ADCs (83). They suit hypoxic tumors, such as DLL3-expressing SCLC, via selective low-pH cleavage (84), although they can be unstable at plasma pH (84) (85)—for example, pH-sensitive linkers in sacituzumab tirumotecan improved ORR in NSCLC, although hepatotoxicity needs monitoring (86). Emerging PEGylation strategies enhance stability (87), with 2024 data showing potential in KRAS-mutated lung cancer (36). Reduction-dependent linkers (e.g., disulfides) are cleaved by tumor glutathione, which suits lung cancer’s redox environment (88). They work well in B7-H3 ADCs but risk premature reduction and systemic toxicity (89)—for example, early c-Met data show intracranial responses (90). Minor types, such as β-glucuronidase-dependent linkers, remain limited and largely exploratory in lung cancer ADCs (91).

Although cleavable linkers dominate the 476 linkers, non-cleavable types (lysosomal, chelation, stable) remain key in lung cancer design (92). They rely on lysosomal degradation for release, offering high stability but lower efficiency (78). Their advantage is reduced off-target toxicity and ILD, making them suitable for prolonged targets like HER2, though limited in low-internalization tumors (93). Limited use of non-cleavable linkers was observed, with a clear preference for cleavable release. This highlights the value of non-cleavable linkers in improving stability and reducing toxicity in heterogeneous tumors. A typical example is the lysosome-dependent maleimide linker, which provides durability in trastuzumab emtansine for HER2-positive NSCLC (94). Recent site-specific conjugation has improved pharmacokinetics and enhanced efficacy/safety in 2025 EGFR-mutated trials (95). Other types, such as chelation for radioactive agents, offer high stability but remain limited and mainly exploratory in lung cancer (36).

Payload distribution prioritizes high-potency cytotoxics to maximize tumor killing while addressing heterogeneity and resistance. DNA topoisomerase I inhibitors dominate (mainly DXd and SN-38), providing anti-resistance and bystander effects in NSCLC through DNA breaks and topoisomerase I-DNA stabilization. Combined with cleavable linkers for selective release, they produce sustained responses, particularly in low-expression and resistant tumors (96)—for example, TROPION-Lung01 validated DXd ADCs in pretreated NSCLC (97), with enhanced efficacy in EGFR-mutated and HER2-mutated cases in DESTINY-Lung trials (98). Tubulin inhibitors rank second (MMAE/DM4 main). They induce mitotic arrest via microtubule interference, leading to cell cycle arrest and apoptosis, which is unique in lung cancer. Their use is limited, often with non-cleavable linkers to improve stability and reduce toxicity in advanced disease, and they show potential in brain metastases (93). Mechanistic benefits are confirmed. MMAE drove an ORR of ~35% in the LUMINOSITY trial for c-Met high NSCLC (58). DM4 requires management of ocular and neurotoxicity (93). Overall, these data emphasize the need to balance safety with Topo I inhibitors in heterogeneous lung cancer (99).

In lung cancer ADC trials, tumor biomarkers are central to patient screening and efficacy prediction (93). Biomarker assessment was integrated in 85.84% of trials, with prognosis, induction, diagnosis, and prediction functions dominating nearly all studies; companion diagnostics accounted for approximately 26%, while early detection and risk assessment were relatively limited. This multidimensional strategy highlights the field’s shift toward an integrated biomarker framework, serving not only for tumor diagnosis and induction but also for efficacy prediction and prognostic evaluation, enabling precise patient stratification (100). Most applications remain in preclinical or early-phase stages, facing challenges in delivery and toxicity. The literature highlights the potential of non-cytotoxic stimulants in lung cancer, particularly when combined with PD-1/PD-L1 inhibitors to improve the tumor microenvironment. However, phase I/II studies are still needed to confirm safety (101). Overall, this distribution reflects a balanced approach to potency, immune evasion, and synergy, providing a solid foundation for optimizing precision medicine in heterogeneous lung cancer.

In clinical trials of ADCs for lung cancer, biomarkers have become the core pillar for patient screening and efficacy prediction (102). In this study, up to 85.84% of trials integrated biomarker assessment, with prognosis, induction, diagnosis, and prediction functions dominating nearly all studies. Companion diagnostics accounted for ~26%, while early detection and risk assessment were limited. This multidimensional combination strategy highlights the field’s strategic tilt toward an integrated biomarker framework, serving not only for diagnosis and induction but also for efficacy prediction and prognostication, enabling precise patient stratification (100). These observations are consistent with current clinical practice. Most trials use antigen expression (e.g., high HER2/TROP2/c-Met) as predictive markers, guiding patient selection via IHC or NGS with gradient approaches for low-expression tumors (103). Companion diagnostics further strengthen prediction, as HER2-mutated or c-Met-high patients achieve higher ORR and PFS (58). Prognosis and induction functions aid heterogeneity monitoring and early resistance detection (58). However, limitations include a lack of standardized thresholds, platform heterogeneity, and uncertainty in low-expression and heterogeneous tumors (11). Future composite markers such as ctDNA combined with AI scoring will be key to enhancing ADC precision in lung cancer (101).

This study provides a comprehensive analysis of global ADC data for lung cancer across SCLC and NSCLC. The extensive coverage and detailed target-linker-payload analysis offer key insights for optimization and reveal important trends such as dual-target strategies. ADCs show clear potential in lung cancer, particularly in advanced disease. However, translation faces challenges, including target complexity and immune escape. While manufacturing complexity and high costs limit broader application, future efforts should focus on optimizing design, reducing off-target effects, developing multi-target combinations, and improving production. The goal is to advance ADCs from laboratory to clinical use, ultimately providing more effective and safer options for lung cancer patients. Limitations exist. First, Trialtrove reliance introduces bias, omissions, and lagging updates. Geographic bias toward developed regions limits underdeveloped/diverse applicability. Future integration of platforms and real-world data, supplementing unregistered reports/abstracts, may strengthen efficacy/safety.

A key focus is structural design, where variability in designs, regimens, criteria, and testing causes substantial heterogeneity, making comparisons and evaluations of outcomes such as toxicity, resistance, and survival particularly challenging—a critical barrier to translation. Future efforts should standardize protocols and conduct meta-analyses to enhance reliability. Although ADCs hold great promise for lung cancer, manufacturing complexity, high costs, and off-target effects currently limit their applicability. The key limitations of this study include reliance on the Trialtrove database (potential omissions and lag), lack of meta-analysis, and geographic bias toward developed regions. Addressing scalability, adverse event management, and real-world ethics will be crucial for broader adoption. These obstacles do not diminish the findings but rather highlight opportunities; overcoming them will unleash the full potential of ADCs against refractory lung cancer and advance targeted therapy.

## 5 Conclusion

This study analyzed global ADC trial data for lung cancer, including SCLC and NSCLC, by type and phase. Extensive data and detailed classification of targets, linkers, and payloads provide insights for optimizing ADC design in lung cancer and identify key clinical trends, such as the potential of dual-target strategies. ADC therapy shows promise in lung cancer treatment, particularly for advanced tumors, and has demonstrated clinical benefit. However, the clinical translation process faces challenges, including the complexity of target selection and the mechanisms of immune escape. Additionally, the complexity of manufacturing processes and high treatment costs limit the widespread application of this therapy. Future research should focus on optimizing ADC design, reducing off-target effect risks, developing multi-target and combination treatment strategies, and improving large-scale production technologies. These advancements will help advance ADC therapy from the laboratory to broader clinical applications, ultimately providing lung cancer patients with more effective and safer treatment options.

## Data Availability

The datasets presented in this study can be found in online repositories. The names of the repository/repositories and accession number(s) can be found in the article/**Supplementary Material**.
